# Conventional and Organic Honeys as a Source of Water- and Ethanol-Soluble Molecules with Nutritional and Antioxidant Characteristics

**DOI:** 10.3390/molecules26123746

**Published:** 2021-06-19

**Authors:** Magdalena Polak-Śliwińska, Małgorzata Tańska

**Affiliations:** 1 Department of Commodity Science and Food Analysis, Faculty of Food Science, University of Warmia and Mazury, Plac Cieszyński 1, 10-718 Olsztyn, Poland; m.polak@uwm.edu.pl; 2 Department of Food Plant Chemistry and Processing, Faculty of Food Science, University of Warmia and Mazury, Plac Cieszyński 1, 10-718 Olsztyn, Poland

**Keywords:** honey, phenolic compounds, DPPH test, ORAC test, sugar profile, Maillard reaction product, ascorbic acid

## Abstract

The benefits of natural honeybee products (e.g., honey, royal jelly, beeswax, propolis, beevenom and pollen) to the immune system are remarkable, and many of them are involved in the induction of antibody production, maturation of immune cells and stimulation of the immune system. The type of plants in the geographical area, climatic conditions and production method have a significantly influence on the nutritional quality of honey. However, this variability can influence consumer liking by the sensory attributes of the product. The aim of this work was to compare the most popular honeys from Poland in terms of nutritional value, organoleptic properties and antioxidant activity. In the study, five varieties of honey (honeydew, forest, buckwheat, linden and dandelion) from conventional and organic production methods were tested. The nutritional characteristics of honey samples included acidity, content of water, sugars, vitamin C, HMF and phenolics (total and flavonoids), while honey color, taste, aroma and consistency were investigated in the organoleptic characteristics. The antioxidant activity was determined in water- and ethanol-soluble honey extracts using DPPH and ORAC tests. The results showed that organoleptic and nutritional characteristics of popular Polish honeys differ significantly in relation to plant source and production method. The significant effect of honey variety on the content of HMF, saccharose and phenolics, as well as acidity and antioxidant capacity were noted. The impact of variety and variety × production method interaction was significant in the case of the content of vitamin C, glucose and fructose. A visible difference of buckwheat and forest honeys from other samples was observed. The highest content of total phenolics with antioxidant activity based on the SET mechanism was found in buckwheat honeys, while forest honeys were richer in flavonoids.

## 1. Introduction

Honey is one of the most popular natural products with unique properties due to the presence of different groups of health-promoting substances [1,2]. It represents a complex mixture of nutrients and bioactive compounds [3,4,5,6,7,8,9]. The flowers used by bees, as well as regional beekeeping practices influence the composition and quality of honey [1]. In addition, the production and supply of honey depends on the climatic variability of the environment or the geographical location of the country, so most of the production occurs in a few regions of the world, such as North and South America, Asia and Europe and India and China, which have become the main exporters of honey in Europe and North America [10,11,12,13]. In organic agriculture, honey bees are the basis of honey production, which is the final commercially available product [1,10]. For honey bees, some activities of beekeepers are prohibited by EU regulations, creating welfare challenges compared to conventional honey production [1,2]. Adulteration related to processing, packaging and improper beekeeping practices such as overheating, feeding bees during honey production, adding different sugars and/or syrups after production and improper labelling are also eliminated [1,10,12]. The global pandemic in 2020 caused the demand and supply of various products to change significantly [11,12]. The demand for honey has increased significantly worldwide due to its nutritional properties and consumer interest in healthy living and immune boosting [12]. Honey has been used for many years to treat many conditions, e.g., chronic colds, coughs, bronchial infections, asthma, ulcers, constipation [1,2,4]. It can contribute to human health and nutrition, e.g., during convalescence, by providing energy to muscles. These beneficial effects are attributed to its anti-inflammatory, antioxidant and antimicrobial potential [4]. Beekeepers, together with local authorities, are making efforts to restore the pace of honey production and supply chain [12]. Many researchers have addressed the quality and composition of honey from different sources in their work [1,7,8,9]. Some of the most important honey properties tested in assessing its quality are moisture content, pH value, electrical conductivity, ash content, free acidity, diastase activity, sucrose and reducing sugars content and hydroxymethylfurfural (HMF) content [7,8,9]. Important from the consumer’s point of view are its organoleptic properties. Honey comes in different colors, and its taste and texture also differ depending on the flower nectar from which it was produced (nectars from many different flowers) [1,2,6]. Raw honey is of the highest ecological quality and is considered to be 100% pure [1]. In Poland, as in other countries, the demand for honey is steadily increasing due to its nutritional and medicinal values (botanical richness and natural biodiversity). However, reports on the physicochemical and sensory quality of available honey vary from location to location [1,2,5,6,7,9], especially since during the pandemic it was most readily available through internet sales without the possibility of product verification. In addition, there are adulterated honeys whose quality is difficult to ascertain. It is therefore important that retailers and consumers are well informed about the quality of the honey they buy. Lately, extracts from honey bee products are becoming increasingly popular in food, drug and cosmetics industries. While honey is more frequently diluted in water, pollen and bee bread are usually extracted using ethanol. Literature data showed that extracts from honey bee products obtained with use other solvents (e.g., ethanol, ethyl acetate) than water are generally richer in phenolic compounds and possess higher antioxidant and antimicrobial activity. The dependence between the solvent used to extract of bioactive compounds and their antioxidant properties were confirmed in samples of propolis and bee pollen [8]. However, the previous studies of bioactive compounds extractability with different solvents have not been carried out on honey samples.

The aim and novelty of this study was to investigate the effect of variety and production method on nutritional, organoleptic and antioxidant properties of Polish honeys from different areas of Poland. The main focus of the study was the solubility in water and ethanol, as well as the antioxidant activity of phenols contained in honey, which can support the immune system.

## 2. Results and Discussion

### 2.1. Nutritional Characteristics of Honeys

Honey moisture content depends on the environmental conditions and the beekeeper manipulation in the harvest period [14]. Honey with high water content is more likely to ferment, making the preservation and storage more difficult. The results of water content in the tested honey samples are shown in Table 1. This parameter in conventional honeys ranges from 14.8% to 19.8%, while in organic honeys from 16.1% to 19.9%, which is in the acceptable range according to the EC Directive 2001/110 [15]. The lowest content of water was found in honeydew honeys (average value of 15.4%), while the highest (average value of 19.1%)—in dandelion honeys. Linden honey was characterized by similar values of this quality factor, regardless of the production method. The difference found in this case was at the level of approx. 2.5%. On the other hand, the greatest disproportion was noticed in the case of forest honeys and it amounted to approx. 12%.

The results of potential acidity measured by titration with sodium hydroxide solution are presented in Table 1. The highest values of the parameter were found for conventional buckwheat and forest honeys (47 and 43 mEq/kg, respectively). The lowest acidity was determined in conventional linden honey (32 mEq/kg). The largest difference (approx. 19%) between organic and conventional production methods was noticed for the linden honeys. Honey pH is affected by the conditions during processing and storage, which also influences texture, stability and shelf-life. The low pH value and the high acidity correspond to the longer honey shelf life because these conditions do not favor microbial growing. On the other hand, high acidic values may indicate the presence of undesirable fermentation, especially in honey with higher water content [16]. The results of the determination of active acidity (pH measurement) are shown in Table 1. High values of this indicator were measured for both dandelion honeys, conventional linden honey and organic honeydew honey (pH in the range of 4.8–4.9). The rest of honeys were more acidic, and the pH value was in the range of 3.6–4.3. The lowest pH value among the analyzed honey samples was determined for conventional buckwheat honey. It was found that the greatest difference between the pH of organic and conventional production methods concerned linden honeys. In this case, organic honey was characterized by a pH value lower by approx. 12% than conventional honey. On the other hand, the method of honey production and origin had no effect on the active acidity of forest and dandelion honeys.

The nutritional quality of food is based on vitamin C analysis as one of the most frequently used indicators of quality [9]. It may strengthen honey’s antibacterial activity [17]. The processing and storage of honey, as well as its botanical origin influence vitamin content and its antioxidant capacity [18]. This vitamin has been found in almost all types of honey [19]. Honey is not an important source of vitamin C, as is described in the present study as well as other literature. In our study (Table 1), honeys with high amounts of vitamin C were: honeydew (3.86–4.17 mg/kg), forest (2.56–2.77 mg/kg), buckwheat (2.51–2.73 mg/kg), lime (2.17–4.47 mg/kg) and dandelion (3.75–4.11 mg/kg). Ciulu et al. [20,21] used RP-HPLC method to simultaneously determine five water-soluble vitamins (B2, B3, B5, B9 and C) in 25 Sardinian honey samples of 10 different botanical origins, in addition to three monofloral honeys (acacia and linden) from northern Italy. The amount of water-soluble vitamin including vitamin C found in honey was also quite low: the overall concentration of all analytes did not exceed 40 mg/kg. The content of vitamin C was low (over 5 mg/kg) and invariant with respect to the origin of honey. The correlation of analyzed vitamins to the botanical origin of the samples may be a useful tool to determine the origin of honeys.

However, honey may contain undesirable compounds like furan derivatives (it may lead to toxicity), which create during a heat treatment facilitating the filtration process, reduce viscosity, delay the crystallization and prevent fermentation during honey processing [22]. The Maillard reaction is a chemical reaction between amino acids and reducing sugars and occurs during cooking conditions, as well as in food storage at low temperature [23,24,25]. Hydroxymethyl-furfural (5-hydroxy-2-furaldehyde, HMF) is one of many important Maillard reaction products and the evaluation of HMF level is a well-known procedure to investigate the quality of honey (an indicator of quality different food products). Due to its adverse effects on human health, like cytotoxic, mutagenic, genotoxic and carcinogenic consequences, the HMF level is limited for some foods such as molasses and honey [22,26,27,28,29,30]. HMF is absent in fresh honeys immediately stored by bees and tends to increase during processing and/or aging of the product; therefore, the HMF content is widely recognized as a parameter of honey sample freshness [31,32]. In our study (Table 1), the levels of HMF were distributed over a wide range of concentrations (6.05–54.25 mg/kg) in honey samples. The HMF content was the lowest in honeydew honeys (10.85 mg/kg in conventional and 6.05 mg/kg in organic). These results are in agreement with the results published by other authors [33,34,35,36,37,38,39,40]. The exceeded amount of HMF was detected in the conventional and organic buckwheat honey samples (48.42 and 54.25 mg/kg, respectively). These values were higher than requirements (<40 mg/kg) established by Polish and international standards for honey [41,42,43]. It can be assumed that these honeys were probably adulterated by invert syrup or they were exposed to excessive heating.

Fructose, glucose and sucrose, as the most important sugars analyzed in the honey samples, are related to those present in the nectar foraged by bees to make honey in such a way that identification of source is possible [44]. Mean sugar content with the corresponding standard deviation, sum of reducing sugars and fructose/glucose ratio (F/G) are shown in Table 2. The two reducing sugars fructose and glucose were detected in all honey samples. Sucrose, however, was not detected in 6 samples. Fructose and glucose represented the largest portion of honey (in case of organic honeys average 72.06 g/100 g and 70.37 g/100 g in the case of conventional honeys). Sucrose formed the lowest portion (in case of organic honeys average 1.29 g/100 g and 0.94 g/100 g in case of conventional honeys). The proportion of fructose to glucose fluctuates considerably but there is always more fructose than glucose in fresh nectar obtained from flower [45,46,47]. The results of presented study agree with these findings. Reducing sugars, mainly fructose and glucose, stand for the largest portion of honey composition, while sucrose content was the lowest in all honey samples. These results are in conformity with the standard requirements [48]. This proves adequate honey treatment, good maturity, energy value and high viscosity [49,50,51,52].

Fructose levels not were significantly different in the honey samples. Its level varied from 37.01 to 42.75 g/100 g.

The lowest fructose content was in organic dandelion honey, while the same honey variety from conventional method was characterized by the highest amount of this sugar. Fructose levels were significantly higher in all samples compared with glucose. Most of the physical and nutritional characteristics of honey depends on the content of this sugar [53]. Mendes et al. [54] found fructose to be the largest portion of 50 Portuguese honey samples evaluated. Golob and Plestenjak [47] found that the differences in fructose and glucose mass fraction among different types of honey are significant. The maximum value of fructose (42.75 g/100 g) in other study was similar to maximum values 40.1 and 40.6 g/100 g in two honey samples from Spain evaluated by Mateo and Bosch-Reig [55]. Fructose range was different, indicating the variety of floral sources from which the honey samples originated.

The second important sugar in honey after fructose is glucose. Its levels were significantly different in the honey samples. The organic honeydew honey contained the lowest amount of glucose (27.17 g/100 g), while the organic linden honey has the highest amount (36.47 g/100 g). The glucose mean value (31.80 g/100 g for conventional honeys and 31.27 g/100 g for organic honeys) of the present study is similar with the results reported by Golob and Plestenjak [47] and Gomez Barez et al. [56]. These researchers have shown the glucose levels for Slovene (29.4 g/100 g) and Spanish (29.2 g/100 g) honeys.

The sucrose content in the studied honeys was ranging from 0 g/100 g in most samples to 4.57 g/100 g in organic linden honey. Sucrose content was the lowest among the 3 tested sugars, which were determined in honey samples as a result of enzyme invertase action, which breaks down the disaccharide molecule of sucrose in the nectar into the monosaccharides, glucose and fructose during the process of ripening of honey [49,57,58]. However, sucrose, which is not highly soluble in honey’s water, can provide information about adulteration and botanical origin of the honey [35,36,53]. In the present study, the mean sucrose was 1.21 g/100 g in organic honeys and 0.94 g/100 g in conventional honeys—similar to that found by Perez-Arquillue et al. [59] and Golob and Plestenjak [47] in Spanish and Slovakian honeys, respectively. In turn, it is lower than the 3.3 g/100 g and 3.0 g/100 g sucrose reported by Abu-Tarboush et al. [60] and Yilmaz and Yavuz [61] in Saudi and Turkish honeys, respectively. Perez-Arquillue et al. [59] reported sucrose content below 1 g/100 g in several Spanish honey samples of different botanical origins. Absence of sucrose in six samples may be due to these honeys being obtained from nectars, which contain just fructose and glucose as a result of total conversion of sucrose into these monosaccharides before their secretion nectars [62,63]. Only fructose and glucose in some of their analyzed honey samples from Indonesia and Nigeria were detected by White et al. [64] and Agwu et al. [46]. The high level of sucrose in honeys may be a result of unripe honey as it was collected from uncapped honeycomb [46,49,63].

An indication of the ability of honey to crystallize is fructose to glucose ratio (F/G), which is used to typify honey samples from different origins, it is the aforementioned indicator, the tendency of crystallization. The organic honeydew honey with low F/G ratio (0.73) crystallized after its collection and storage at room temperature. F/G ratio in organic honey samples ranged between 0.73–0.95 and in conventional honey samples ranged between 0.74–0.83. The highest F/G ratio was observed in organic linden honey (0.95). A high or low ratio signifies liquid or crystallized honey, respectively [60]. Similar results were found with Litchi honeys analyzed by Suryanarayana et al. [65]. The viscosity of honey can be examined by its texture. The wide range of F/G ratio in present study may be indicative of the variety of floral sources from which the honey samples originated. Mateo and Bosch-Reig [55] obtained a similar range of F/G ratio for Spanish honey samples (0.99–1.40 g/100 g). While Perez-Arquillue et al. [59] reported narrower range (1.06–1.13 g/100 g) in rosemary honeys, which displayed a remarkable low variation among unfloral samples.

### 2.2. Organoleptic Characteristics of Honeys

**Figure 1:**
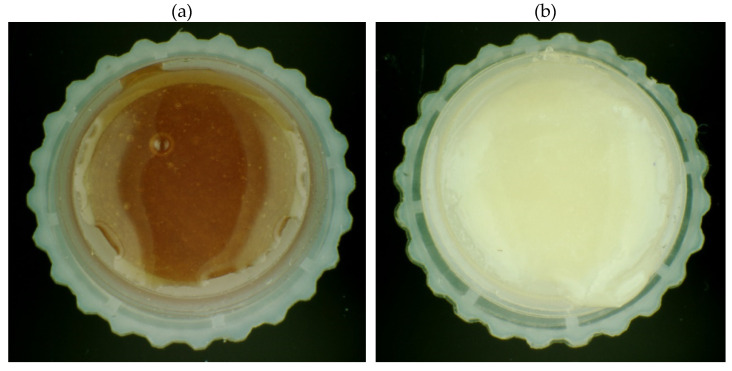
Photo of conventional buckwheat honey (**a**) and ecological forest honey (**b**).

The results of the organoleptic evaluation are presented in Table 3. On their basis, it was found that the color of organic and conventional honeys does not differ significantly in the case of the linden, forest and honeydew honeys. On the other hand, color differences were observed within buckwheat and dandelion honeys. Differences in color of tested honey samples show selected photos (Figure 1). The taste of all nectar honeys was characterized as spicy with varying degrees of intensity. Honeydew and forest honeys were described as mild, with notes of resin and pine needles in the scent. Nectar honeys, on the other hand, were characterized by a subtle scent of flowers from which the main pollen was obtained. The consistency of the honey, depending on the individual characteristics of the variety, showed a fine-crystalline structure for linden, dandelion and forest honeys produced conventionally. The consistency of the honeydew honey was medium-grained crystallization, while the organic forest honey contained coarse-grained crystals. In buckwheat honeys, the crystal size was not specified because they were in the form of a strained honey.

The results of the organoleptic evaluation carried out in the study indicated a certain differentiation of the tested honey samples, both in terms of variety (honeydew, forest, buckwheat, linden, dandelion) and the method of its production (organic, conventional). The final color of honey is created by the content of pollen and pollutants from the environment. It confirms the possibility of unconscious creation of honey features uncharacteristic for the variety. Different organoleptic characteristics such as consistency, taste and aroma of the same varieties from organic and conventional honeys may result from the degree of crystallization, type of fruit, harvest time or environmental conditions. Moreover, it is indicated that the length of honey storage has an impact on the content of essential oils, therefore buckwheat, honeydew, dandelion and linden honeys may differ in aromas within the same variety.

Popov-Raljić et al. [66] also presents significant differences in the organoleptic characteristics in the case of monofloral (acacia) and multifloral (meadow) honeys from Serbia. They reported that variation in honey properties is a consequence of different types of honey, different geographical and botanical origin, chemical composition, weather conditions, beekeeping and other factors. Silvano et al. [67] analyzed the physicochemical parameters and the sensory properties in honeys from different regions of Buenos Aires province. They suggest that could be possible to classify honeys according to the geographic origin based on the physicochemical parameters, while the sensory properties were not good predictors.

### 2.3. Content of Water- and Ethanol-Soluble Phenolics in Honeys

The total contents of water-soluble phenolics in the tested honeys are presented in Table 4. They were from 6.84 mg/100 g for conventional linden honey to 206.25 mg/100 g for conventional buckwheat honey. In turn, ethanol-soluble phenolics constituted from 2.92 mg/100 g to 216.78 mg/100 g for the same honeys, respectively. The lowest content of phenolics among the tested organic honeys was found in dandelion honey (water and ethanol extracted compounds up to 17 mg/100 g). No relationship was observed between the origin of the honey and the total content of phenolics. In total, 11 organic and 11 conventional honey samples from Poland were investigated by Halagarda et al. [9]. Authors determined the phenolic profiles of several varieties of Polish honey and their correlation with various factors influencing the quality of honey. They also verified the impact of production method (conventional/organic) and the pollen content on these profiles.

The total content of flavonoids in the tested honeys was clearly lower (Table 4). The water-soluble flavonoids ranged from 2.44 mg/100 g for conventional buckwheat honey to 13.61 mg/100 g for organic honeydew honey. On the other hand, when extracting these compounds with ethanol, their content was 1.6–3.0 times lower. The lowest content of flavonoids among the tested organic honeys was found in linden honey, while the lowest content of flavonoids among conventional honeys was found in buckwheat honey. No relationship was observed between the origin of the honey and the content of flavonoids. However, a relationship was demonstrated between the solvent used and the total content of flavonoids in honey. Higher values were found in the case of extracting flavonoids with water. Pauliuc et al. [68] described the physicochemical characteristics of honey (raspberry, mint, rape, sunflower, thyme and multifloral) produced in Romania and reported that the flavonoid content was influenced not by botanical origin but year.

Many studies have shown that honey has valuable activity against respiratory pathogens [69], including viruses that cause several viral diseases [70,71,72,73]. In addition, honey also possesses anti-inflammatory properties and is recognized as an immune booster, which complements it as an effective means of reducing the severity of viral diseases [74,75,76]. Most of the medicinal and especially nutritional properties of honey have been linked to the antioxidant phenolic compounds contained in it [77]. The diverse chemical structures including phenolic acids and polyphenols (e.g., flavonoids) are characterized of phenolic compounds in honey. The most abundant phenolic acids are gallic acid, chlorogenic acid, syringic acid, vanillic acid, *p*-coumaric acid, *p*-hydroxybenzoic acid and caffeic acid, while the most abundant flavonoids in honey are apigenin, chrysin, quercetin, luteolin, kaempferol, galangin, genistein, pinocembrin and pinobanksin [77,78]. A study of 10 monofloral and multifloral honeys showed that the antioxidant activities, based on their phenolic content, of some monofloral honeys were higher compared to multifloral honeys, whereas other monofloral honeys showed lower antioxidant activities [79,80].

### 2.4. Antioxidant Activity of Water- and Ethanol-Soluble Compounds in Honeys

The results of the determination of the antioxidant activity of the tested honeys against DPPH radicals are presented in Table 5. In the case of water-soluble compounds, the DPPH radical scavenging capacity was within the range of 0.64 mM TE/100 g for organic dandelion honey to 1.58 mM TE/100 g for conventional buckwheat, while in the case of ethanol-soluble compounds, this parameter ranged from 0.60 to 1.06 mM TE/100 g for the same honeys, respectively. It was found that linden honey was characterized by the lowest antioxidant activity among conventional honeys, while buckwheat honey was characterized by the highest antioxidant activity among organic honeys. The strongest antioxidant activity for buckwheat honey was also confirmed by Dżugan et al. [69]. The difference between conventional and organic honeys ranged from 8% (honeydew honeys) to 30% (forest honeys). In general, water-soluble compounds of honeys showed stronger antioxidant properties in the test with the DPPH radical. Moreover, in most cases, conventional honeys showed a higher DPPH radical scavenging activity. The only exception was linden honey, in the case of which the tendency was opposite.

The results of the ORAC test are presented in Table 5. It was shown that with the use of ethanol for the extraction of honey antioxidants, the oxygen radical absorption capacity was in the ranged from 0.86 mM TE/100 g for organic dandelion honey to 3.72 mM TE/100 g for forest honey from the same production method. However, in the case of the use of water, the values of this parameter were less differentiated and ranged from 1.98 mM TE/100 g for organic honeydew honey to 3.40 mM TE/100 g for conventional linden honey. Among organic honeys, buckwheat and honeydew honeys showed the highest oxygen radical absorption capacity.

The results of the research showed a strong correlation between the content of total phenolics and the antioxidant activity determined in the test with DPPH radical. The correlation coefficient for all tested honeys was 0.83; for honeys from conventional method production r = 0.81, while for organic honeys r = 0.93. There was a moderate correlation between the antioxidant activity determined in the ORAC test and the total phenolics content in organic honeys, where r = 0.57. However, the content of flavonoids in honeys does not affect their antioxidant activity.

The correlations between antioxidant activity and total concentration of phenolics were confirmed for strawberry tree honeys from Italy [81] and floral origin honeys from Romania [82]. The antioxidant activities based on the free radical scavenging, reducing power, and bleaching inhibition were investigated for the three commonly used honeys in Malaysia (tualang, gelam and acacia honey) by Chua et al. [83]. The authors showed that antioxidant capacity of the honey samples correlated with the content of total phenolics and flavonoids. The total flavonoid content of honeys strongly correlated with the three antioxidative tests, while the total phenolics content not correlated with the DPPH test. In turn, no significant correlation between phenolic contents and antioxidant activity of raw honeys from Algeria was found in work of Ahmed et al. [7].

The differences in antioxidant activity of water-soluble compounds were relatively more visible among honeys analyzed using the DPPH test, while in the ORAC test—ethanol-soluble compounds. The differences may be a result of the distinct mechanisms of these tests. Hydrogen atom transfer (HAT) mechanism is studied in ORAC test and single electron transfer (SET) mechanism is typical for DPPH test [84]. Our study shows that the HAT mechanism generally predominated for honey compounds regardless of the method of their extraction used.

### 2.5. Comparison of Honeys in Terms of Variety and Production Method

**Figure 2:**
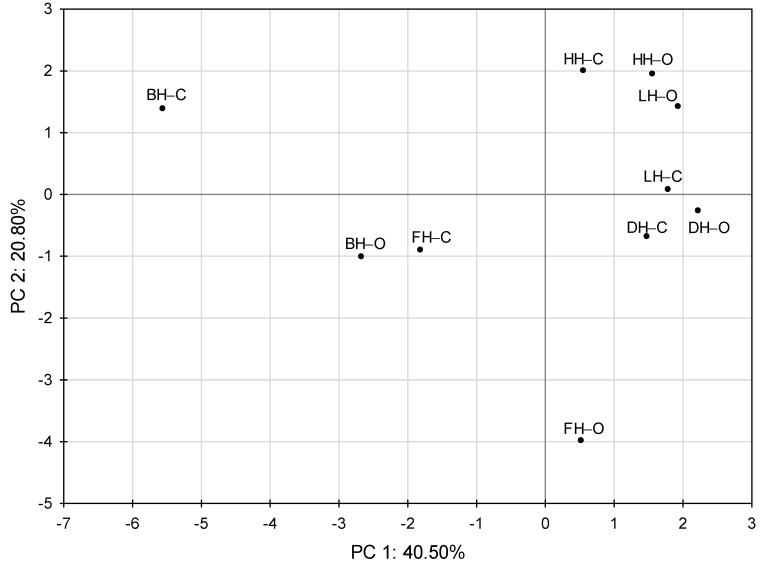
PCA score plot showing differentiation of tested honey samples. Abbreviations of honey samples are explained in Table 7. HH—honeydew honey, FH—forest honey, BH—buckwheat honey, LH—linden honey, DH—dandelion honey, C—conventional production method, O—organic production method.

Principle component analysis (PCA) score plot (Figure 2) showed the relationships between honey samples. The first two main components explained 61.30% of the total variance. The PC1 explained 40.50% of the variance and PC2 explained next 20.80% of the variance. A visible separation of buckwheat and forest honeys and an overlapping of other samples were observed. The method of production also resulted in a shift of scores of buckwheat and forest honeys. The results of the study showed that honeydew, linden and dandelion honeys characterized by higher content of vitamin C and lower acidity. In turn, buckwheat and forest honeys were distinguished by much higher content of total phenolics and total flavonoids, respectively. Dżugan et al. [69] has also shown that buckwheat and honeydew honeys exhibit higher values of antioxidant activity and contain more phenolic compounds than dandelion and multifloral honeys.

Effect of variety and production method on tested parameters in honeys is shown in Table 6. The highest effect of honey variety on HMF and saccharose contents (>90% of explained variance) was noted. The variety was also most decisive for acidity, phenolics content and antioxidant activity of honey (42.4%–73.3% of explained variance). It was found a significant impact of variety and variety × production method interaction on content of vitamin C, glucose and fructose in honey. These properties were only slightly production method dependent. Moderate effect (32.9% of explained variance) of honey production method on content of flavonoids was noticed. The summarized effect of interactions of both tested factors was the highest for fructose content (46.1%).

## 3. Materials and Methods

### 3.1. Honey Samples

The samples of honeys were bought in the same year directly from certified producers and stored at −18 ± 2 °C until use. The study comprised 10 fresh (up to one month after production) honey samples (*n* = 3) of 5 varieties. Additionally, each variety was represented by an organic and a conventional sample (Table 7). The samples came from the following regions of Poland: Podlaskie, (samples Nos. FH-O, BH-O, DH-O), Podkarpackie (samples Nos. HH-O, LH-O), Silesia (samples Nos. HH-C, FH-C, DH-C), Warmia and Masuria (samples Nos. BH-C, LH-C). The pollen frequency in honey samples was not examined. However, plant source was declared by beekeepers who tested honey samples every year in certified laboratory (>60% of a specific pollen type).

### 3.2. Chemicals and Reagents

Analytical-grade reagents such as aluminum chloride, sodium nitrite, metaphosphoric acid, Carrez solutions, KH_2_PO_4_ (ABChem, Olsztyn, Poland), 2,2’-Azobis(2-amidinopropane) dihydrochloride (AAPH), 2,2-diphenyl-1-picrylhydrazil (DPPH), gallic acid, apigenin, fluorescein, Folin-Ciocalteau reagent and Trolox, ethanol, hydroxy methyl furfural (HMF), ascorbic acid, fructose, glucose and saccharin (Sigma-Aldrich, Saint Louis, MO, USA), sodium carbonate and sodium hydroxide (POCH, Gliwice, Poland) were used. Other applied reagents, i.e., methanol, acetonitrile was of the highest purity (chromatography-grade) available and purchased from the Sigma-Aldrich Chemical Company (Saint Louis, MO, USA). Deionized water was obtained from HLP 5 deionizer (Hydrolab, Gdańsk, Poland).

### 3.3. Determination of Water Content

The water content was determined by the refractometric method specified in the Regulation of the Ministry of Agriculture and Rural Development of 14 January 2009 [85]. About 5 g of mixed honey were placed in a test tube and brought to a liquid state in a water bath at a temperature of 35–45°C. Using a rod, a few drops of honey were placed on the lower prism of the refractometer, spread over the entire surface and covered with the upper prism. The measurement was carried out and the refractive index was read from the refractometer scale to four decimal places. Based on the refractive index, the percentage of the extract and the water content in percent by weight were read using the table included in the above-mentioned Regulation of the Ministry of Agriculture and Rural Development [85].

### 3.4. Determination of Honey Acidity

Free acidity and pH were measured according to AOAC method 962.19 [86]. Free acidity was determined by the titrimetric method. 10 g of honey were dissolved in 75 mL deionized water, and this solution was titrated with NaOH 0.1 M solution until the pH reached 8.5. The pH was determined by a HI 9125 pH-meter, equipped with an HI 1083B electrode (Hanna Instruments, Cluji-Napoca, Romania).

### 3.5. Determination of Vitamin C Content

Isolation of HMF from honey sample was based on the procedure described by León-Ruiz et al. [18]. Five grams of homogenized honey were dissolved in 25 mL of 2% (*w/v*) HPO_3_, filtered through a paper filter and prior to injection in the HPLC system, filtered again through a 0.2 µm cellulose acetate (CA) membrane filter. HPLC analyses were carried out on an LC-10A system (Shimadzu, Kyoto, Japan) equipped with a diode array detector (DAD). Chromatographic separation was achieved on a Phenomenex^®^ Synergy 4u Hydro-RP 80 A column (250 × 4.6 mm I.D., particle size 5 µm) with precolumn Phenomenex^®^ Security Guard Cartiges AQ C18 (4 × 3.0 mm) in isocratic mode, with a mobile phase of 0.1 M KH_2_PO_4_ in water at pH 2.4. The detection wavelength was set at 246 nm. The injection volume was 50 µL and the flow rate was 0.5 mL/min. Stock standard solution (1 mg/mL) was prepared by dissolving 10 mg of vitamin C in 10 mL of 2% (*w/v*) HPO_3_ solution. The content of vitamin C has been calculated using the external standard method. Calibration curve was prepared for different concentrations of vitamin C standards in the range of 0.1–25 mg/L. The method characterized a good sensitivity with a detection limit (LOD) 0.1 mg/L and quantification limit (LOQ) value 0.25 mg/L. Recovery mean of ascorbic acid in honey was 99% (accuracy of the method).

### 3.6. Determination of HMF Content

HMF was measured by HPLC-DAD according to the AOAC method [87,88] with some modifications. Honey samples were prepared after a cleaning procedure. For this purpose, 10 g of honey sample was diluted to 50 mL with demineralized water and after clarification by Carrez I (K_4_Fe(CN)_6_) and Carrez II (Zn(CH_3_COO)_2_) solutions it was filtered through 0.45 µm nylon membrane filter and injected into an HPLC-RP system. The HPLC column was a Phenomenex^®^ Synergy 4u Hydro-RP 80 A, 250 × 4.6 mm, particle size 5 µm, fitted with a Phenomenex^®^ Security Guard Cartiges AQ C18, 4 × 3.0 mm to protect analytical column. Chromatography analyses were carried out with a Shimadzu LC-10A HPLC device (Shimadzu, Kyoto, Japan) with a diode array detector (SPD–M 20A). The HPLC conditions were the following: isocratic mobile phase, at 4% acetonitrile in deionized water; flow rate 1.0 mL/min; temperature of the column 25 °C and injection volume 50 µL. Monitoring of the analytes were made by using a DAD detector at 280 nm wavelength. Stock solutions HMF were prepared as 1000 ppm in acetonitrile. Standard solutions of HMF (concentration: 0.1; 0.5; 1; 5; 10 ppm) were prepared by diluting of these stock solutions with mobile phase.

### 3.7. Determination of Sugars Content

The HPLC method described by Myhara et al. [89] was used to identify and quantify the main sugar profile (fructose, glucose, sucrose) in honey samples. For each sugar a standard (4.0 g/L solution in deionized water) was prepared. Honey samples (0.25 g) were dissolved in 5 mL of deionized water. Three replicates were prepared for each honey sample. Samples were cleaned by using a Sep-Pak plus^®^C18 cartridge (Waters Corporation) by the SPE method and a 0.2 mm filter (Satorius-Minister^®^ NML). The cartridge was activated by methanol and rinsed with water before use. Two ml were used to wash the cartridge materials. The samples were freshly prepared and immediately analyzed HPLC-NP (LC-10A Shimadzu, Kyoto, Japan) with a refractive index detector (RID). As the column was used LC-NH_2_ column (Agilent Zorbax NH_2_, 250 × 4.6 mm, 5 µm). The refractive index detector was used to monitor the analytes. The temperature of the column and the refractometer was adjusted at 40 °C. The separation was achieved with a mobile phase acetonitrile/water (85:15) solvent system at a flow rate of 1.5 mL/min. Sugar standard was injected into HPLC prior to any honey sample (50 μL) injections. The sugar profile in the sample was identified and quantified by the software program LC Solution (Shimadzu, Kyoto, Japan). The quantitative and qualitative interpretation of the obtained chromatograms was carried out on the basis of the comparison of the retention time and the size of the area of glucose, fructose and sucrose peaks in standard samples of known concentration, and retention time and the size of the analyte peak area in the test samples. The fructose/glucose ratio was also calculated.

### 3.8. Evaluation of Honey Organoleptic Properties

The organoleptic evaluation was performed by 30 untrained panelists of both sexes, with ages ranging between 21 and 60 years. The samples were coded with three-digit numbers and were served at room temperature (5 g of honey in plastic cups). Mineral water and salt crackers were supplied as palate cleansers between sample evaluations. The honey samples were divided in two sessions, and five samples per session were evaluated. The methodology used according to Piana et al. [90] the following evaluations: color, taste, aroma and consistency of honey.

### 3.9. Determination of Content of Water- and Ethanol-Soluble Phenolics

The honey solutions were prepared as follows: 10 g of honey was weighed with an accuracy of 0.001 g, dissolved in water or ethanol (in 50 mL) and stirred for 30 min at room temperature (20 ± 2 °C) using a shaker. Then the sample was centrifuged for 10 min (16,000 rpm) on the 5810R-type centrifuge (Eppendorf, Hamburg, Germany).

The content of total phenolics was determined spectrophotometrically with the use of Folin-Ciocalteu reagent according to the method described by Singleton et al. [91], with some modifications. The color reaction was carried out by adding to the 0.5 mL honey solution 0.5 mL of Folin-Ciocalteu reagent diluted in deionized water (1:2, *v/v*), 3.5 mL of 14% sodium carbonate and completed to 10 mL with deionized water. After mixing, the solution was left in the dark at room temperature for 60 min. Absorbance was then measured against the reagent sample at a wavelength of 720 nm using the FLUOstar Omega microplate reader (BMG LABTECH, Offenburg, Germany). The content of total phenolics was expressed as mg of gallic acid equivalent (GAE) in 1 g of honey.

The content of total flavonoids was determined spectrophotometrically according to the methodology of Chua et al. [83], with some modifications. The color reaction was carried out by adding to the 1 mL honey solution 2 mL water, 0.3 mL 5% solution of sodium nitrite and 0.6 mL 10% solution of aluminum chloride. A flavonoid–aluminum complex was formed after 10 min of incubation time at 25 °C. The solution was centrifuged for 10 min (16,000 rpm) on the 5810R-type Eppendorf Centrifuge, and absorbance was measured at 415 nm using the FLUOstar OMEGA microplate reader. The content of flavonoids was expressed as mg of quercetin equivalent (QE) in 1 g of honey.

### 3.10. Determination of Antioxidant Activity of Water- and Ethanol-Soluble Compounds

The antioxidant activity of water and ethanolic solutions of honey was determined by the DPPH Radical Scavenging Assay and the Oxygen Radical Absorbance Capacity (ORAC) assay. The DPPH test was carried out according to Yang et al. [92], with some modifications. Two mL of a DPPH solution (0.2 mmol/L in methanol) was added to 0.5 mL of honey solution. The mixture was shaken and incubated in the dark at room temperature for 30 min. Absorbance was measured at 517 nm against methanol using a FLUOstar Omega microplate reader. The antioxidant activity was expressed as mM Trolox equivalent (TE) per 100 g of honey.

The ORAC test was carried out according to the method described by Huang et al. [93], with some modifications. 25 µL of honey solutions and pure solvent (blank sample) were placed in wells of a black 96-well plate with 150 μL of 10 nM fluorescein (in 75 mM phosphate buffer, pH 7.4). The mixtures were pre-incubated at 37°C for 15 min, and then 25 μL of 153 mM AAPH (in 75 mM phosphate buffer, pH 7.4) was added. The fluorescence intensity was measured automatically by the FLUOstar Omega microplate reader (excitation at 485 nm, emission at 540 nm) every 1 min for 6 h. The antioxidant activity was expressed as mM TE per 100 g of honey.

### 3.11. Data Analysis

All analyses were conducted in triplicate and the data expressed as mean ± standard deviation. The results were evaluated statistically using analysis of variance (ANOVA) followed by the Tukey test. The impact of plant source and production method on studied parameters of honey was determined using two-way analysis of variance. All calculations were performed using Statistica 12.5 PL software (StatSoft, Kraków, Poland) at *p* ≤ 0.05 significance level.

## 4. Conclusions

Despite the small number of samples in the study and its pilot character, it was observed that the organoleptic and nutritional features of honeys popular in Poland differed significantly depending on the plant source and the production method. This may indicate the influence of many factors on the honey production process, as well as the unique character of the product, which obliges further extensive research. Generally, the organic honeys were characterized by darker color with higher yellowness, higher content of vitamin C and flavonoids and lower content of HMF compared to the conventional ones. In contrast, the content of total phenolics and antioxidant activity were slightly higher in conventional honeys, regardless of the extraction solvent employed. All tested honeys were generally good quality. However, the HMF content of 2 samples from 10 analyzed honey samples were above the requirements established by Polish and international standards for honey. This excessive HMF contents is a concerning point for public health and the national authority needs to increase its supervision on the production of honey. According to new literature data, honey has protective effects for the treatment of various disease conditions (e.g., diabetes mellitus, respiratory, gastrointestinal, cardiovascular, nervous systems, cancer treatment) because of the presence many antioxidants. We found that organoleptic properties of honey could be considered as the first indicator suggesting the greater antioxidant potential of honey. Generally, honeys with light color, delicate aroma and not intense taste were characterized by lower content of total phenolics with lower antioxidant activity. In turn, the high concentration of these compounds was specific for brown and spice honeys. The study also showed that extraction solvent is important factor that influence on phenolic compounds content and antioxidant activity of obtained extracts. Most water-soluble extracts were richer in flavonoids than ethanol-soluble ones, whereas the total phenolic compounds content was higher in ethanolic extracts from some organic honeys (e.g., forest, linden, dandelion) compared to extracts prepared with water. The antioxidant activity of honey samples in ORAC test was more differentiated than in DPPH test. Water-soluble honey compounds were characterized by higher DPPH radical scavenging than ethanol-soluble compounds. In turn, in ORAC test ethanolic extracts from honeydew samples and conventional forest honey had higher antioxidant activity than those obtained with water.

## Figures and Tables

**Table 1:** Physicochemical parameters of honey samples (mean value and standard deviation).

Symbol	Water Content (%)	Acidity (mEq/kg)	pH (−)	Vitamin C Content (mg/kg)	HMF Content (mg/kg)
HH-C	14.8 ± 0.5^a^	37 ± 0.2^ab^	4.53 ± 0.05^ab^	3.86 ± 0.17^e^	10.85 ± 0.11^c^
HH-O	16.0 ± 0.2^a^	34 ± 0.5^a^	4.85 ± 0.04^ab^	4.17 ± 0.14^f^	6.05 ± 0.22^a^
FH-C	18.1 ± 0.1^a^	43 ± 0.2^ab^	4.03 ± 0.08^ab^	2.56 ± 0.24^b^	28.15 ± 0.15^f^
FH-O	19.8 ± 0.5^a^	42 ± 0.4^ab^	4.10 ± 0.12^ab^	2.77 ± 0.23^c^	9.21 ± 0.17^b^
BH-C	18.5 ± 0.4^a^	47 ± 0.6^b^	3.63 ± 0.08^a^	2.73 ± 0.42^c^	48.42 ± 1.12^h^
BH-O	19.2 ± 0.3^a^	40 ± 0.2^ab^	4.00 ± 0.06^ab^	2.51 ± 0.15^b^	54.25 ± 1.10^i^
LH-C	17.9 ± 0.5^a^	32 ± 0.7^a^	4.95 ± 0.04^b^	2.17 ± 1.05^a^	22.81 ± 0.11^e^
LH-O	17.5 ± 0.3^a^	38 ± 0.1^ab^	4.35± 0.07^ab^	4.47 ± 1.01^g^	18.51 ± 0.34^d^
DH-C	19.8 ± 0.5^a^	33 ± 0.2^a^	4.87 ± 0.05^ab^	3.75 ± 0.11^d^	29.84 ± 0.21^g^
DH-O	18.5 ± 0.2^a^	35 ± 0.5^a^	4.79 ± 0.06^ab^	4.11 ± 0.22^f^	28.75 ± 0.25^f^
Mean	18.01 ± 1.60	38.10 ± 4.86	4.41 ± 0.46	3.31 ± 0.84	25.68 ± 15.99
C.V.	8.86	12.77	10.35	25.38	62.27
Mean-C	17.82 ± 1.84	38.40 ± 6.47	4.40 ± 0.56	3.01 ± 0.75	28.01 ± 13.61
C.V.	10.34	16.84	12.80	24.92	48.60
Mean-O	18.20 ± 1.50	37.80 ± 3.35	4.42 ± 0.39	3.61 ± 0.90	23.35 ± 19.41
C.V.	8.23	8.85	8.81	24.88	83.10

C.V.—coefficient of variation (%), a,b,c—mean values with the same letter are not significantly different at *p* ≤ 0.05. Abbreviations of honey samples: HH—honeydew honey, FH—forest honey, BH—buckwheat honey, LH—linden honey, DH—dandelion honey, C—conventional production method, O—organic production method.

**Table 2:** Sugar profile in honey samples (mean value and standard deviation).

Symbol	Glucose Content (g/100 g)	Fructose Content (g/100 g)	Saccharose Content (g/100 g)	Reducing Sugars (g/100 g)	Glucose/Fructose Ratio
HH-C	30.86 ± 0.15^d^	38.56 ± 1.10^d^	1.20 ± 0.00^b^	69.42 ± 0.00^d^	0.80 ± 0.00^f^
HH-O	27.17 ± 1.14^a^	37.32 ± 1.15^b^	1.50 ± 0.00^c^	64.49 ± 0.01^a^	0.73 ± 0.00^a^
FH-C	34.51 ± 0.42^i^	41.5 ± 2.14^h^	0.00 ± 0.00^a^	76.01 ± 0.01^j^	0.83 ± 0.00^g^
FH-O	32.07 ± 1.23^g^	42.17 ± 2.13^i^	0.00 ± 0.00^a^	74.24 ± 0.00^g^	0.76 ± 0.00^c^
BH-C	28.71 ± 0.24^b^	38.71 ± 1.40^e^	0.00 ± 0.00^a^	67.42 ± 0.02^c^	0.74 ± 0.00^b^
BH-O	31.55 ± 0.25^e^	40.53 ± 1.05^g^	0.00 ± 0.00^a^	72.08 ± 0.09^e^	0.78 ± 0.00^d^
LH-C	33.17 ± 0.11^h^	39.77 ± 2.00^f^	3.51 ± 0.11^d^	72.94 ± 0.00^f^	0.83 ± 0.00^g^
LH-O	36.47 ± 2.01^j^	38.47 ± 2.01^c^	4.57 ± 0.15^e^	74.94 ± 0.10^i^	0.95 ± 0.00^h^
DH-C	31.75 ± 0.21^f^	42.75 ± 1.21^j^	0.00 ± 0.00^a^	74.50 ± 0.07^h^	0.74 ± 0.00^b^
DH-O	29.11 ± 0.12^c^	37.01 ± 1.12^a^	0.00 ± 0.00^a^	66.12 ± 0.01^b^	0.79 ± 0.00^e^
Mean	31.54 ± 2.78	39.68 ± 2.00	1.08 ± 1.68	71.22 ± 4.07	0.80 ± 0.06
C.V.	8.82	5.04	155.45	5.72	8.16
Mean-C	31.80 ± 2.22	40.26 ± 1.82	0.94 ± 1.53	72.06 ± 3.56	0.79 ± 0.05
C.V.	6.97	4.52	162.07	4.95	5.78
Mean-O	31.27 ± 3.51	39.10 ± 2.20	1.21 ± 1.99	70.37 ± 4.78	0.80 ± 0.09
C.V.	11.22	5.63	163.54	6.79	10.69

C.V.—coefficient of variation (%), a,b,c—mean values with the same letter are not significantly different at *p* ≤ 0.05. Abbreviations of honey samples: HH—honeydew honey, FH—forest honey, BH—buckwheat honey, LH—linden honey, DH—dandelion honey, C—conventional production method, O—organic production method.

**Table 3:** Organoleptic properties of honeys.

Symbol	Color	Taste	Aroma	Consistency
HH-C	greenish-brown	gentle, sweet	weak, spicy-resinous	medium-grained crystallization
HH-O	tea	slightly resinous, mild, sweet	spicy-resinous	dense liquid, few medium-grained crystals
FH-C	light brown	gentle, noticeable sharp aftertaste	resinous, pine needles	fine-grained crystals
FH-O	light brown	not very intense	weak, resinous	coarse-grained crystals
BH-C	shades of yellow	intense, spicy	similar to the scent of buckwheat flowers	thick, patchy liquid
BH-O	brown	sweet, spicy	intense, buckwheat flowers	thick, patchy liquid
LH-C	light yellow	delicate, almond, slightly spicy	linden flowers, slightly intense	fine-grained crystallization
LH-O	light cream	sweet, spicy	strong, linden flowers	fine-grained crystallization
DH-C	yellow-green	sweet, mild, noticeable slight sharpness	dandelion flowers, not very intense	dense, greasy
DH-O	light yellow	sweet, slightly spicy	faint, dandelion flowers	fine-grained crystallization

Abbreviations of honey samples: HH—honeydew honey, FH—forest honey, BH—buckwheat honey, LH—linden honey, DH—dandelion honey, C—conventional production method, O—organic production method.

**Table 4:** Content of water- and ethanol-soluble phenolics in honey samples (mean value and standard deviation).

Symbol	Total Phenolics Content (mg GAE/100 g of Honey)		Total Flavonoids Content (mg QE/100 g of Honey)	
	Water-Soluble	Ethanol-Soluble	Water-Soluble	Ethanol-Soluble
HH-C	41.08 ± 1.19^c^	28.08 ± 2.70^c^	4.48 ± 0.19^bc^	1.49 ± 0.47^ab^
HH-O	58.82 ± 5.42^d^	30.84 ± 1.41^c^	6.08 ± 0.82^cd^	2.06 ± 0.12^b^
FH-C	81.40 ± 3.73^e^	70.23 ± 8.45^d^	6.33 ± 0.16^cd^	1.95 ± 0.27^ab^
FH-O	16.46 ± 8.22^a^	22.16 ± 1.49^b^	13.61 ± 0.04^e^	4.90 ± 0.23^d^
BH-C	206.25 ± 8.51^g^	216.78 ± 2.37^e^	2.40 ± 0.12^a^	1.54 ± 0.08^ab^
BH-O	93.64 ± 3.85^f^	82.35 ± 5.65^d^	5.64 ± 0.12^cd^	2.92 ± 0.31^c^
LH-C	6.84 ± 3.35^a^	2.92 ± 1.49^a^	3.82 ± 0.04^ab^	1.32 ± 0.00^a^
LH-O	22.72 ± 5.48^b^	29.19 ± 1.22^c^	2.75 ± 0.16^ab^	1.29 ± 0.04^a^
DH-C	12.52 ± 8.13^a^	29.25 ± 3.49^c^	3.66 ± 0.51^ab^	1.68 ± 0.04^ab^
DH-O	10.42 ± 7.72^a^	16.42 ± 2.56^b^	7.32 ± 0.39^d^	3.05 ± 0.43^c^
Mean	55.02 ± 61.39	52.82 ± 62.38	5.61 ± 3.23	2.22 ± 1.13
C.V.	111.58	118.10	57.60	50.74
Mean-C	69.62 ± 81.89	69.45 ± 85.82	4.15 ± 1.42	1.60 ± 0.24
C.V.	117.63	123.57	34.31	14.79
Mean-O	40.41 ± 35.21	36.19 ± 26.44	7.08 ± 4.02	2.84 ± 1.35
C.V.	87.12	73.05	56.73	47.52

C.V.—coefficient of variation (%), a,b,c—mean values with the same letter are not significantly different at *p* ≤ 0.05. Abbreviations of honey samples: HH—honeydew honey, FH—forest honey, BH—buckwheat honey, LH—linden honey, DH—dandelion honey, C—conventional production method, O—organic production method.

**Table 5:** Antioxidant activity of water- and ethanol-soluble compounds in honey samples (mean value and standard deviation).

Symbol	DPPH (mM TE/100 g of Honey)		ORAC (mM TE/100 g of Honey)	
	Water-Soluble	Ethanol-Soluble	Water-Soluble	Ethanol-Soluble
HH-C	1.06 ± 0.23^cd^	0.80 ± 0.05^ab^	2.42 ± 0.08^ab^	3.18 ± 0.05^cd^
HH-O	0.97 ± 0.08^bcd^	0.71 ± 0.06^ab^	1.98 ± 0.06^a^	2.93 ± 0.07^c^
FH-C	0.95 ± 0.06^bcd^	0.82 ± 0.03^ab^	2.91 ± 0.04^bc^	3.72 ± 0.04^d^
FH-O	0.67 ± 0.10^a^	0.68 ± 0.01^ab^	2.55 ± 0.05^ab^	1.87 ± 0.01^b^
BH-C	1.58 ± 0.01^e^	1.06 ± 0.09^c^	2.86 ± 0.01^bc^	1.98 ± 0.03^b^
BH-O	1.13 ± 0.08^d^	0.89 ± 0.02^bc^	2.99 ± 0.06^bc^	2.98 ± 0.02^c^
LH-C	0.65 ± 0.04^a^	0.62 ± 0.03^a^	3.40 ± 0.05^c^	1.92 ± 0.02^b^
LH-O	0.73 ± 0.00^ab^	0.65 ± 0.04^a^	2.31 ± 0.01^ab^	1.45 ± 0.02^ab^
DH-C	0.82 ± 0.09^abc^	0.67 ± 0.01^a^	2.12 ± 0.02^a^	1.08 ± 0.01^a^
DH-O	0.64 ± 0.01^a^	0.60 ± 0.01^a^	2.02 ± 0.04^a^	0.86 ± 0.03^a^
Mean	0.92 ± 0.29	0.75 ± 0.14	2.56 ± 0.47	2.20 ± 0.96
C.V.	31.64	19.14	18.47	43.61
Mean-C	1.01 ± 0.35	0.79 ± 0.17	2.74 ± 0.49	2.38 ± 1.06
C.V.	34.82	21.55	17.92	44.63
Mean-O	0.83 ± 0.21	0.71 ± 0.11	2.37 ± 0.42	2.02 ± 0.93
C.V.	25.71	15.66	17.59	45.97

C.V.—coefficient of variation (%), a,b,c—mean values with the same letter are not significantly different at *p* ≤ 0.05. Abbreviations of honey samples: HH—honeydew honey, FH—forest honey, BH—buckwheat honey, LH—linden honey, DH—dandelion honey, C—conventional production method, O—organic production method.

**Table 6:** Effect of variety and production method (% of explained variance) on tested parameters in honeys.

Tested Parameter	Variety	Production Method	Variety × Production Method	Other Factors
Water content	43.8	ns.	ns.	ns.
Acidity	52.5	ns.	15.6	ns.
pH	49.3	ns.	ns.	ns.
HMF content	90.5	2.4	7.1	ns.
Vitamin C content	55.7	13.9	30.4	ns.
Glucose content	67.3	1.0	31.7	ns.
Fructose content	44.6	9.3	46.1	ns.
Saccharose content	97.6	0.7	1.7	ns.
Content of water-soluble phenolics	73.3	6.2	19.2	ns.
Content of ethanol-soluble phenolics	68.8	7.8	22.2	ns.
Content of water-soluble flavonoids	55.7	22.2	19.3	ns.
Content of ethanol-soluble flavonoids	42.4	32.9	21.2	ns.
DPPH test for water-soluble compounds	72.6	10.3	9.6	ns.
DPPH test for ethanol-soluble compounds	68.8	8.6	ns.	ns.
ORAC test for water-soluble compounds	50.4	14.1	17.2	ns.
ORAC test for ethanol-soluble compounds	68.1	3.7	23.8	ns.

ns.—effect not significant, two-way analysis with Wilks test, *p* ≤ 0.05.

**Table 7:** Origin of honey samples.

Symbol	Plant Source	Production Method	Origin
HH-C	Honeydew	Conventional	Apiary Jakubiec (Silesia, Silesian Foothills)
HH-O		Organic	Apiary Eko Bałon (Podkarpacie, Niżna Łąka)
FH-C	Forest	Conventional	Apiary Jakubiec (Silesia, Silesian Foothills)
FH-O		Organic	Apiary Sznurowski (Podlasie, Nowogród)
BH-C	Buckwheat	Conventional	Apiary Pucer (Warmia and Masuria)
BH-O		Organic	Apiary Sznurowski (Podlasie, Nowogród)
LH-C	Linden	Conventional	Apiary Pucer (Warmia and Masuria)
LH-O		Organic	Apiary Eko Bałon (Podkarpacie, Niżna Łąka)
DH-C	Dandelion	Conventional	Apiary Jakubiec (Silesia, Silesian Foothills)
DH-O		Organic	Apiary Sznurowski (Podlasie, Nowogród)

## Data Availability

The data presented in this study are available on request from the corresponding author. The data are not publicly available due to a large number and variety of analyses.

## Sample Availability

Not available.
